# 

**Published:** 1897-03

**Authors:** 


### BOOKS RECEIVED.

A System of Practical Medicine. By American authors. Edited by Alfred Lee Loomis, M. D., Late Professor of Pathology and Practical Medicine in the New York University, and William Gilman Thompson, M. D., Professor of Materia Medica, Therapeutics and Clinical Medicine in the New York University. To be completed in four imperial octavo volumes, containing from 900 to 1,000 pages each, fully illustrated in colors and in black. Vol. I. Infectious diseases. Just ready. Vol. II. Diseases of the respiratory and circulatory systems, and of the blood and kidneys. In press. Vol. III. Diseases of the digestive system, of the liver, spleen, pancreas and other glands; gout, rheumatism, diabetes and other constitutional diseases. In active preparation. Vol. IV. Diseases of the nervous system and of the muscles; diseases of doubtful origin, insolation, Addison's disease, etc. In active preparation. Per volume, cloth, $5.00; leather, $6.00; half-morocco, $7.00. Philadelphia and New York: Lea Brothers & Co., Publishers.

Twentieth Century Practice. An international encyclopedia of modern medical science. By leading authorities of Europe and America. Edited by Thomas L. Stedman, M. D., New York City. In twenty volumes. Volume X. Diseases of the nervous system. New York: William Wood & Co. 1897.

Principles or Guides for a Better Selection or Classification of Consumptives, amenable to high altitude treatment and to the selection of patients who may be more successfully treated in the environment to which they were accustomed previous to their illness. By A. Edgar Tussey, M. D., Adjunct Professor of Diseases of the Chest in the Philadelphia Polyclinic and School for Graduates in Medicine, and Consulting Physician to the central branch of the Y. M. C. A. Gymnasium, Fifteenth and Chestnut streets. Philadelphia: P. Blakiston, Son & Co., 1012 Walnut street. 1896.

Genito-Urinary Surgery and Venereal Diseases. By J. William White, M. D., Professor of Clinical Surgery, University of Pennsylvania, and Edward Martin, M. D., Clinical Professor of Genito-Urinary Diseases, University of Pennsylvania. Illustrated with 243 engravings and seven colored plates. 8vo, pp. xx.—1,061. Philadelphia: J. B. Lippincott Company. London: 6 Henrietta street, Covent Garden. 1897.

A Manual of the Practice of Medicine, prepared especially for students. By A. A. Stevens, A. M., M. D., Lecturer on Terminology and Instructor in Physical Diagnosis in the University of Pennsylvania ; Demonstrator of Pathology in the Woman's Medical College of Pennsylvania ; Physician to St. Agnes's Hospital, to the out-patient department of the Episcopal Hospital and to the Southeastern Dispensary, Philadelphia. Fourth edition, revised and enlarged. Illustrated. Pp. xviii.—511. Philadelphia : W. B. Saunders, 925 Walnut street. 1896.

Elementary Bandaging and Surgical Dressing, with directions concerning the immediate treatment of cases of emergency for the use of dressers and nurses. By Walter Pye, F. R. C. S., late Surgeon to St Mary's Hospital. Revised and in part re-written by G. Bellingham Smith, F. R. C. S., Surgical Registrar, Guy's Hospital. 16mo, pp. 218. Seventh edition. Philadelphia : W. B. Saunders, 925 Walnut street. 1897.

